# Pulmonary metastasis from urothelial carcinoma of the upper urinary tract 29 years after nephrectomy

**DOI:** 10.1186/s40792-017-0293-3

**Published:** 2017-01-31

**Authors:** Koji Kawaguchi, Toshiki Okasaka, Takayuki Fukui, Koichi Fukumoto, Shota Nakamura, Shuhei Hakiri, Naoki Ozeki, Kohei Yokoi

**Affiliations:** Department of Thoracic Surgery, Nagoya University Graduate School of Medicine, 65 Tsurumai-cho, Showa-ku, Nagoya, 466-8550 Japan

**Keywords:** Urothelial carcinoma, Tumor dormancy, Late metastasis, Long-term latency

## Abstract

**Background:**

Late pulmonary metastasis from urothelial carcinoma (UC) of the upper urinary tract is extremely rare.

**Case presentation:**

A 76-year-old man was referred to our hospital due to an abnormal shadow on chest X-ray. He had a history of left nephrectomy with a diagnosis of UC in the renal pelvis 29 years previously. Computed tomography showed a mass lesion in the right middle lobe of the lung that measured 4.9 cm in diameter. A transbronchial biopsy revealed the tumor to be metastatic pulmonary UC, and he underwent right middle lobectomy of the lung.

**Conclusion:**

Long-term postoperative follow-up of patients with UC might be necessary after radical nephrectomy.

## Background

Urothelial carcinoma (UC) of the upper urinary tract is uncommon and accounts for only 5 to 10% of UCs, in contrast with bladder tumors [1]. Some recurrent cases after long-term latency associated with bladder UC have been reported; however, there have been very few cases of late pulmonary metastasis of upper urinary tract UC. We herein report an extremely rare case of pulmonary metastasis from the upper urinary tract UC 29 years after nephrectomy.

## Case presentation

**Fig. 1:**
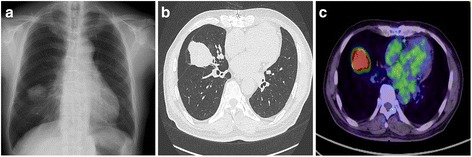
Radiological findings. **a** Chest X-ray shows an abnormal shadow of the right lung. **b** Chest CT shows a mass lesion with a smooth margin in the right middle lobe of the lung. **c** FDG-PET shows that the maximal standardized uptake value is 10.2 in the tumor

**Fig. 2:**
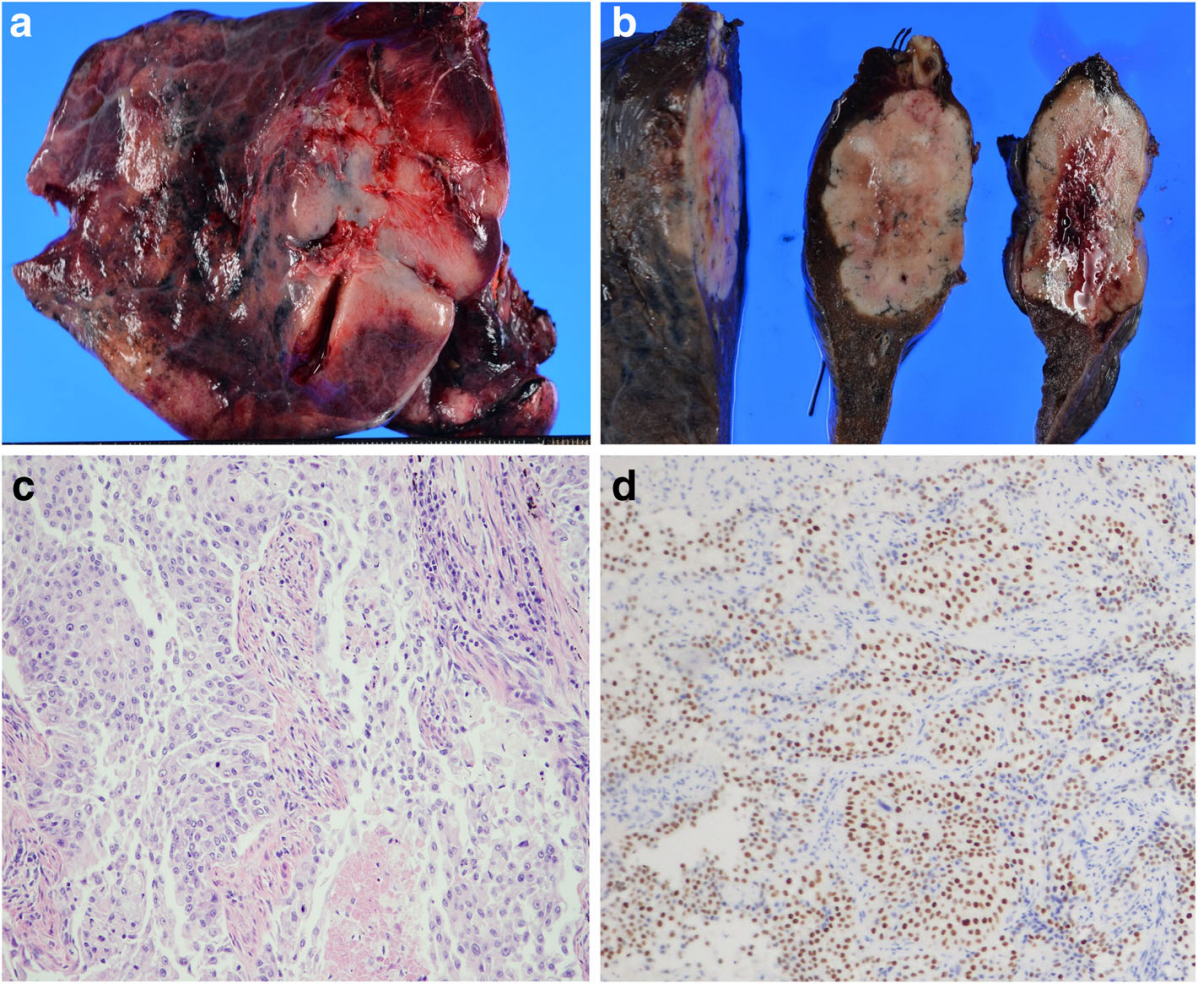
Surgical and pathologic findings. **a**, **b** Macroscopic findings of the surgical specimen are shown. **c** The resected specimen of the lung resembles the previous upper urinary tract urothelial carcinoma. **d** Immunohistochemically, the tumor was positive for GATA3 in cancer cells (×200)

A 76-year-old man who had undergone coronary arterial bypass grafting for ischemic heart disease 3 years previously was referred to our hospital due to an abnormal shadow of the right lung detected on chest X-ray during his usual medical checkup that had not been detected 3 years previously (Fig. 1a). Chest computed tomography (CT) showed a mass lesion with smooth margin in the right middle lobe of the lung that measured 4.9 cm in diameter (Fig. 1b). He also had a history of left nephrectomy with a diagnosis of UC in the renal pelvis (pT2N0M0 pStage II) 29 years previously. 18F-fluorodeoxyglucose-positron emission tomography (FDG-PET) showed a lung mass with a maximal standardized uptake value of 10.2 without any other uptake lesions (Fig. 1c). A transbronchial biopsy of the mass demonstrated histologic features resembling the past renal pelvic carcinoma. The mass was strongly suspected to be a pulmonary metastasis of UC, so he underwent right middle lobectomy of the lung with hilar lymph node dissection (Fig. 2a,b). A pathologic examination of the resected specimen revealed solid nests of tumor cells with clear cytoplasm including finely granular nuclear chromatin and inconspicuous nuclei, findings that were highly consistent with the histology of UC (Fig. 2c). Immunohistochemically, the specimen was positive for GATA-binding protein 3 (GATA3) (Fig. 2d) and negative for thyroid transcription factor-1 (TTF-1). Based on these findings, the tumor was diagnosed as a pulmonary recurrence of the previously resected upper urinary tract UC. The postoperative course was uneventful, and he was alive without disease 5 months after pulmonary resection.

## Conclusions

Upper urinary tract UC is a relatively uncommon disease, accounting for only 5 to 10% of urothelial malignancies. These upper urinary tract cancers are thought to seed tumor cells to the urinary tract, as concurrent bladder cancer is present in 8 to 13% of upper urinary tract UC cases, and recurrence occurs by seeding to urinary bladder in 30 to 51% of the patients [1]. Hall et al. reported that the site of the first relapse of resected upper urinary tract UC was local in the retroperitoneum (tumor bed, soft tissue, or lymph nodes) (9.0%), in the bladder (50.8%), in the remaining upper tract (17.9%), or distantly in the lung, bone, or liver (22.4%) [2]. They also found that the median time to recurrence after curative treatment was 12 months (mean, 23.6 months; range, 1–99 months), and primary tumor stage or surgical procedure performed were important predictors of disease recurrence [2]. Although our patient underwent radical nephrectomy, he had recurrence in the bladder only 1 year after the operation and was treated with bacilli Calmette Guerin therapy and transurethral resection. To our knowledge, only one case of late pulmonary metastases from upper urinary tract UC has been reported. In that case, a pulmonary metastasis with mediastinal lymph adenopathy and brain metastases developed 12 years after radical nephrectomy [3].

Metastatic diseases occasionally occur decades after successful treatment of the primary tumor. It has been proposed that this latency period is due to a clinical phenomenon named tumor dormancy. Several researchers have advocated that the dormancy of such cells might be caused by a non-proliferating state or an arrest in the cell cycle which thus results in a prolonged G0 phase [4]. However, these mechanisms including how these cells can then trigger proliferation have not yet been fully explored.

GATA3 is useful for differentiating metastatic UC from other primary or secondary epithelial neoplasms, including adenocarcinoma and squamous cell carcinoma, as well as sarcoma and melanoma. Morphologically, at least 25% of cases of UC demonstrate foci of squamous or glandular differentiation and overlap with adenocarcinoma, such as intracytoplasmic mucin. In the present case, the tumor was first suspected to be primary lung cancer on CT, without any consideration of pulmonary metastasis of UC. GATA3 is a transcription factor for T cell development and a regulator of estrogen receptor in breast carcinoma. Immunohistochemical staining of GATA3 is now used for the diagnostic support of breast cancer and UC as a tumor marker. Laura et al. reported that GATA3 expression was positive in 88% of invasive upper urinary tract UC and in 80% of pulmonary metastases of UC but was negative in primary pulmonary squamous cell carcinoma [5].

According to the recent guidelines, systemic chemotherapy is the standard treatment for patients with metastatic UC [1]. However, despite initially promising response rates of up to 70%, most patients will eventually experience tumor progression. The median survival following contemporary chemotherapy regimens remains 13–14 months [6]. However, as for the role of surgery in the management of metastatic UC, Matsuguma et al. reviewed 32 patients who underwent pulmonary metastasectomy with curative intent. They reported that the 5-year survival after metastasectomy was 50%, and metastasis exceeding 3 cm in size was proven to be a significant poor prognostic factor on a multivariate analysis [7]. Woo et al. reviewed data from 16 patients who underwent pulmonary metastasectomy with adjuvant chemotherapy and showed that the 5-year overall and disease-free survivals were 65.3 and 37.5%, respectively [8]. They demonstrated a favorable survival rate after repeated pulmonary metastasectomy in five patients, two of whom underwent three resection operations for recurrent disease. Long-term cancer control can be achieved in a subgroup of patients following surgical removal of metastatic UC, but metastasectomy in patients with disseminated metastatic UC remains controversial.

Although late pulmonary metastasis of upper urinary tract UC is extremely rare, physicians should be aware of occurrence and follow up such patients for a long-term period as curative re-treatment may be possible.

## Abbreviations

CT: Computed tomography
FDG-PET: Fluorodeoxyglucose-positron emission tomography
GATA3: GATA-binding protein 3
TTF-1: Thyroid transcription factor-1
UC: Urothelial carcinoma

## References

[1] Rouprêt M, Zigeuner R, Palou J, Boehle A, Kaasinen E, Sylvester R (2011). European guidelines for the diagnosis and management of upper urinary tract urothelial cell carcinomas: 2011 update. Eur Urol..

[2] Hall MC, Womack S, Sagalowsky AI, Carmody T, Erickstad MD, Roehrborn CG (1998). Prognostic factors, recurrence, and survival in transitional cell carcinoma of the upper urinary tract: a 30-year experience in 252 patients. Urology..

[3] Lim JH, Jeon SH, Lee JM, Kim L, Cho JH, Ryu JS (2013). Late onset distant metastatic upper urinary tract urothelial carcinoma mimicking lung adenocarcinoma. Tuberc Respir Dis..

[4] Friberg S, Nystrom A (2015). Cancer metastases: early dissemination and late recurrences. Cancer Growth Metastasis..

[5] Hoang LL, Tacha D, Bremer RE, Haas TS, Cheng L (2015). Uroplakin II (UP II), GATA3, and p40 are highly sensitive markers for the differential diagnosis of invasive urothelial carcinoma. Appl Immunohistochem Mol Morphol..

[6] Lehmann J, Suttmann H, Albers P, Volkmer B, Gschwend JE, Fechner G (2009). Surgery for metastatic urothelial carcinoma with curative intent: the German experience. Eur Urol..

[7] Matsuguma H, Yoshino I, Ito H, Goya T, Matsui Y, Nakajima J (2011). Is there a role for pulmonary metastasectomy with a curative intent in patients with metastatic urinary transitional cell carcinoma?. Ann Thorac Surg..

[8] Han WS, Kim K, Park JS (2012). Result of surgical resection for pulmonary metastasis from urothelial carcinoma. Korean J Thorac Cardiovasc Surg..

